# A pneumatic Bionic Voice prosthesis—Pre-clinical trials of controlling the voice onset and offset

**DOI:** 10.1371/journal.pone.0192257

**Published:** 2018-02-21

**Authors:** Farzaneh Ahmadi, Farzad Noorian, Daniel Novakovic, André van Schaik

**Affiliations:** 1 The MARCS Institute for Brain Behaviour and Development, Western Sydney University, Sydney, New South Wales, Australia; 2 School of Electrical and Information Engineering, The University of Sydney, Sydney, New South Wales, Australia; 3 Central Clinical School, Faculty of Medicine, The University of Sydney, Sydney, New South Wales, Australia; University of Adelaide, AUSTRALIA

## Abstract

Despite emergent progress in many fields of bionics, a functional Bionic Voice prosthesis for laryngectomy patients (larynx amputees) has not yet been achieved, leading to a lifetime of vocal disability for these patients. This study introduces a novel framework of Pneumatic Bionic Voice Prostheses as an electronic adaptation of the Pneumatic Artificial Larynx (PAL) device. The PAL is a non-invasive mechanical voice source, driven exclusively by respiration with an exceptionally high voice quality, comparable to the existing gold standard of Tracheoesophageal (TE) voice prosthesis. Following PAL design closely as the reference, Pneumatic Bionic Voice Prostheses seem to have a strong potential to substitute the existing gold standard by generating a similar voice quality while remaining non-invasive and non-surgical. This paper designs the first Pneumatic Bionic Voice prosthesis and evaluates its onset and offset control against the PAL device through pre-clinical trials on one laryngectomy patient. The evaluation on a database of more than five hours of continuous/isolated speech recordings shows a close match between the onset/offset control of the Pneumatic Bionic Voice and the PAL with an accuracy of 98.45 ±0.54%. When implemented in real-time, the Pneumatic Bionic Voice prosthesis controller has an average onset/offset delay of 10 milliseconds compared to the PAL. Hence it addresses a major disadvantage of previous electronic voice prostheses, including myoelectric Bionic Voice, in meeting the short time-frames of controlling the onset/offset of the voice in continuous speech.

## Introduction

The Bionic Voice source is an electronic prosthesis which substitutes for the voice generation function of the missing vocal folds of a laryngectomy patient [1]. The theory of human speech generation is based on a source-filter model in which a source, (generally a combination of aspiration noise and glottal pulse train generated by vocal folds), is filtered through a resonance model of the vocal tract. In that sense, laryngectomy patients lose the ability to generate and control this voice source signal, while retaining a functional vocal tract filter. The primary aim of designing a Bionic Voice prosthesis is to substitute the missing vocal folds and generate this glottal voice source signal for patients. Hence, Bionic Voice should be distinguished from other voice rehabilitation technologies e.g. voice conversion approaches [2] where patients initially use a voice prosthesis to speak and their generated speech is then modified to sound more natural through post-processing [3].

Two main questions are inherent in designing any Bionic Voice prosthesis: 1) How to provide patients with the ability to naturally control their artificial source of voice as they speak and 2) how to synthesize a (more) natural sounding human voice. While the two questions may be equally important, this research focuses on addressing the first.

The main focus of research in electronic voice prostheses has been Electrolarynx (EL) which uses a (voluntary) manual control of the voice onset, offset and pitch. Despite more than sixty years of research since the invention of the EL, very little progress has been made to reach any substantial improvement in intelligibility or to enhance its robot-like quality [4]. On the other hand, the gold standard of Tracheoesophageal (TE) voice prosthesis [5] and the Pneumatic Artificial Larynx (PAL) devices continue to generate a superior voice quality and outperform the EL both in terms of intelligibility and naturalness [6–13]. Contrary to the EL, both the TE and the PAL enable the patients to control their voice generation naturally using the residual physiology of phonation. The design question then turns into: What contributes to the intuitive control of the TE and PAL devices and how can it be employed to control a Bionic Voice source?

**Fig 1:**
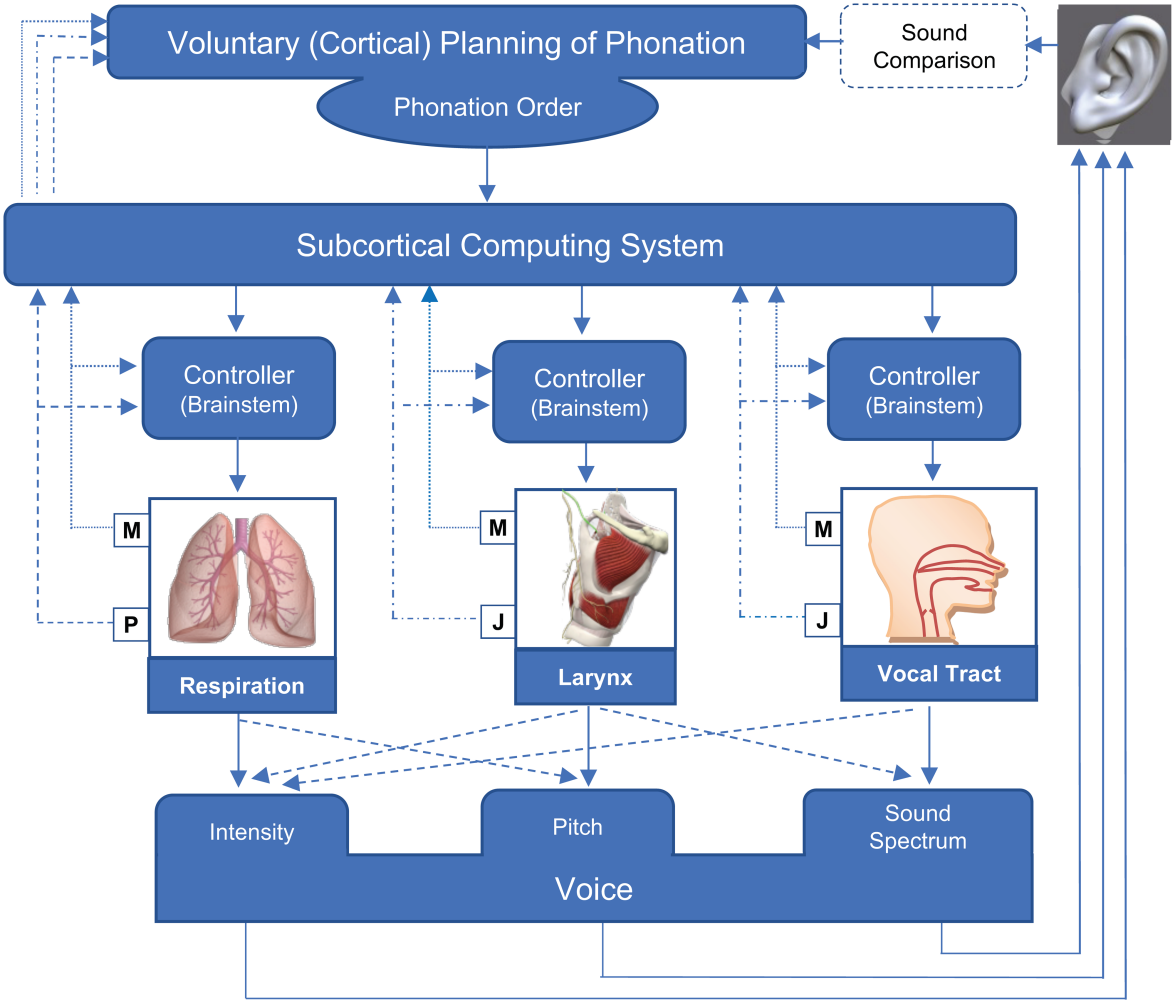
Components of natural control of phonation. The involuntary control system benefits from feedback from the hearing, respiratory, laryngeal and articulation systems. P: Pressure sensing feedback, J: articulator positions sensing feedback, M: Muscle movement sensing feedback (adapted from [16]).

An extensive survey of the physiology of voice control [14, 15], demonstrates that natural control of phonation is largely involuntary [16]. Fig 1 shows a schematic of the physiological components involved in controlling voice generation during speech. During the phonation process, the respiratory, laryngeal and articulatory (vocal tract) subsystems are coordinated by the brain stem. It is well known that the phonation order, initiation, and termination of the speech and in part, pitch variations are voluntarily planned in the cortical (voluntary) region of the brain. An involuntary sub-cortical computing system then uses this control data and leads the mechanism of voice control (e.g. the timing of voice onset/offset inside the words in speech), almost automatically [16].

After the laryngectomy, the patient maintains the articulatory and respiratory systems, however, the mechanism of respiration changes and the patient has to breathe through an opening in the anterior neck (stoma). During natural voice generation, respiratory control coexists with laryngeal control to maintain and control the pressure of exhaled airflow passing through the larynx [17]. In normal speech, respiratory control contributes to the control of pitch (micro-prosody) [18], loudness and specifically stress [19, 20] and plays a significant role in controlling micro-prosody of speech in tonal languages [8, 9].

A significant observation possibly overlooked in designing electronic voice prostheses is that after the loss of larynx, the respiratory system can play a dominant role in controlling voice generation [21, 22]. This is specifically observed in the PAL device, a mechanical voice source, exclusively driven by variations in pressure above and below the missing larynx [21, 22] with a voice quality, superior to the Electrolarynx and comparable to the gold standard of (TE) voice prosthesis [8, 13, 21–23]. Despite its limited prevalence due to its cumbersome design [9] and the unhygienic coupling of the stoma to the mouth [24], the PAL voice has been described as clearer with higher levels of intelligibility, less noise, and greater short-term pitch variations (micro-prosody, at syllable level) when compared to TE speech in multiple trials [6–13, 21, 22].

This advocates the study of the PAL as a non-invasive, yet effective reference model for designing electronic voice prostheses driven exclusively by respiration. This research aims to be the first in this path, proposing a Pneumatic Bionic Voice source as an electronic adaptation of the traditional PAL device and addressing the main shortcomings of the mechanical PAL device. Following the PAL mechanism of voice generation as the reference, the Pneumatic Bionic Voice prostheses are similarly expected to provide a quality comparable or better than the existing gold standard while remaining non-invasive and non-surgical [7–9].

The following, reports the efforts of the authors in modelling the voice onset/offset control of the PAL device and implementing it in an algorithm to control a Pneumatic Bionic Voice prototype in real-time.

## Method

### A pneumatically driven Bionic Voice source

**Fig 2:**
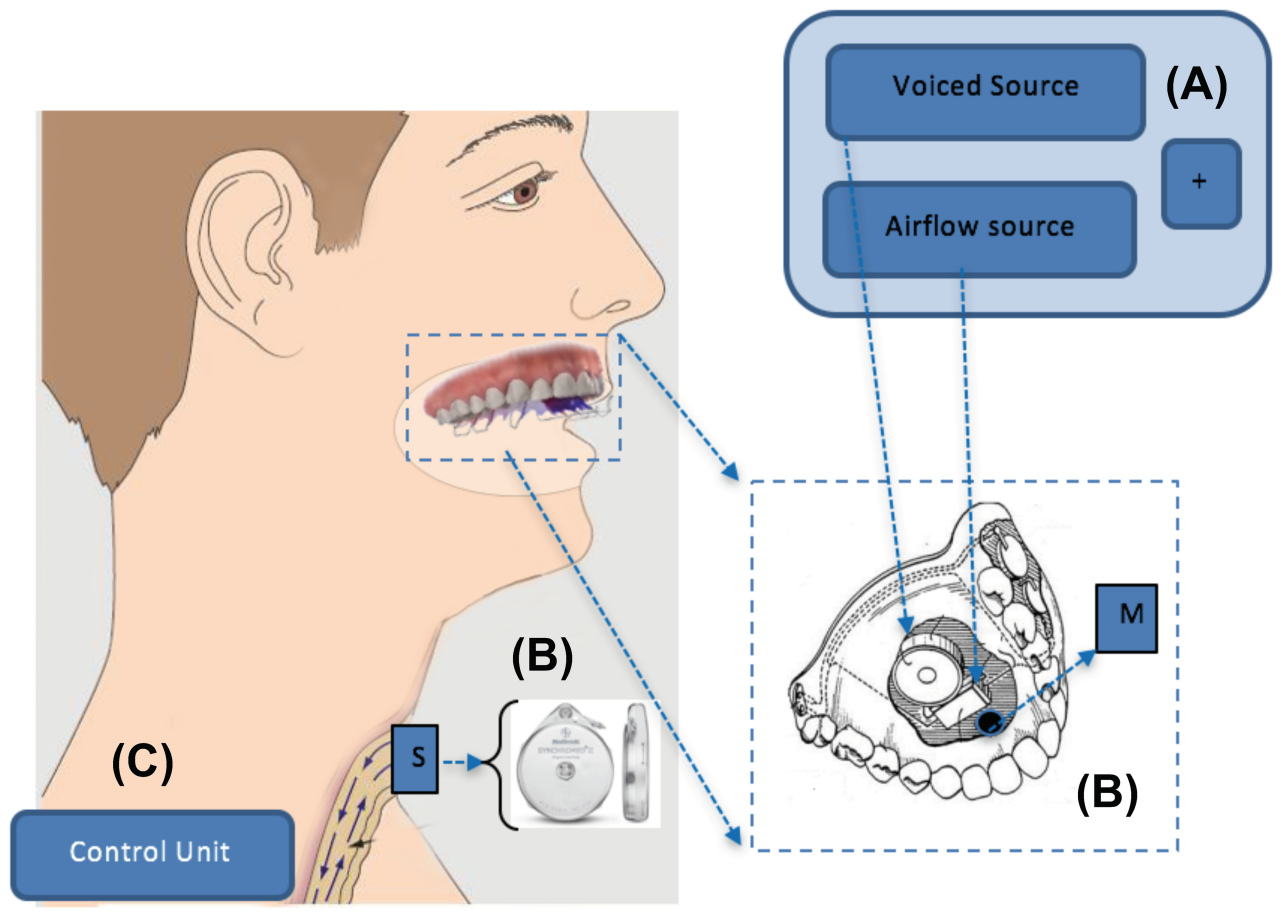
The three components of a Pneumatic Bionic Voice system as a conceptualized device. A) The hybrid voice source, B) the respiratory pressure sensing by S: stoma and M: mouth pressure sensors, C) the Control unit.

The PAL device is a hybrid voice source that provides both voiced and airflow components required for generating voiced and unvoiced speech. As its electronic adaptation, a Pneumatic Bionic Voice source is comprised of three modules (Fig 2):

A) The hybrid electronic voice source to generate voiced and airflow components. The voiced source is generated by a miniature loudspeaker. A simulated PAL (or vocal folds) model provides the voiced component signal to substitute the voice generation of the mechanical reed element of the PAL. The airflow component is generated by a micro-blower and is proportional to the airflow of the stoma. The resulting voiced and airflow components are combined and applied to the vocal tract.

B) A set of pressure sensors to monitor the intra-oral and stoma pressures (the pressure above and below the missing larynx respectively). As a mechanical reed, the PAL is driven by the variations of these two pressures at its input and output sides [25].

C) A control unit which uses the monitored pressure values to predict the onset and offset instances and the pitch of the voiced component together with the magnitude of the added airflow.

The main reported disadvantage of the traditional PAL device is its cumbersome design [9] and the need for a tube to transfer the sound to the vocal tract resulting in an unhygienic direct coupling of the stoma to the mouth [24]. This is addressed in the design of Fig 2 by providing a wireless link between the pressure sensing unit at the stoma and the intra-oral source.

### The PAL mechanism of voice generation in speech

When used in speech as a voice source, the PAL complies with two main hypotheses that underlie a source-filter separation of speech: A) The length of PAL’s resonant tube is small compared to the quarter wavelength of the resonance of the reed. The tube has an acoustic output impedance, much larger compared to the vocal tract [26]. Hence, the PAL reed oscillates close to its natural frequency [27–29]. B) The natural frequency of the reed (100 ± 20Hz) is significantly lower than the vocal tract first formant so, the acoustic coupling of the PAL and the vocal tract can be assumed to be insignificant [30, 31].

With the source-filter separation assumption in place, similar to the vocal folds, the PAL can be modelled in two different modes of operation in speech: when the source undergoes small-amplitude oscillations in voiced speech and 2) when it reacts to large amplitudes of the driving pressure in the transition between voiced/unvoiced speech. The latter is directly relevant in understanding PAL’s mechanism of controlling the voice onset and offset. In both cases, the reed oscillations are driven by the pressure difference between the two sides of the reed *P*_*g*_ = *P*_*s*_−*P*_*m*_ [32, 33] (with pressure inside the mouth *P*_*m*_ and the stoma *P*_*s*_).

The small amplitude, self-oscillation of the PAL reed in voiced speech can be described by a simple lumped element model of a free reed or a single mass-spring model of vocal folds [25, 34, 35]. Increasing the driving pressure (*P*_*g*_), the PAL exits the small amplitude oscillation regime and moves toward a switched behaviour turning the voice on or off. A hysteresis effect is experimentally observed in the voice onset/offset transition of the PAL. Such hysteresis phenomenon is well-known to vocal folds vibrations with a higher value of pressure threshold for the onset of the voice compared to the offset [36, 37]. The voice onset/offset hysteresis is also experimentally observed in the oscillations of the vocal folds in excised larynx [36, 38], the mechanical replica of vocal folds (with or without vocal tract coupling) [30, 39–41] and pneumatically driven mechanical free reeds [42].

Lucero et al. have proven both theoretically [43] and empirically [44] that a mechanical replica of the vocal folds still demonstrates the voice onset/offset hysteresis in the absence of vocal tract coupling. With the source filter separation in place, this framework [43] equally describes the case of an ideal PAL where a mechanical replica of the vocal folds (placed external to the body) substitutes the PAL’s reed [44]. The significance of this configuration is that the use of a vocal fold replica in an ideal PAL is expected to generate a more natural sound compared to a PAL with a simple reed. Hence, implementing the hysteresis behaviour will remain a general requirement for designing Pneumatic Bionic Voice prostheses to approach the performance of an ideal PAL in controlling the voice onset and offset.

### A numerical hysteresis model for the PAL onset/offset control

**Fig 3:**
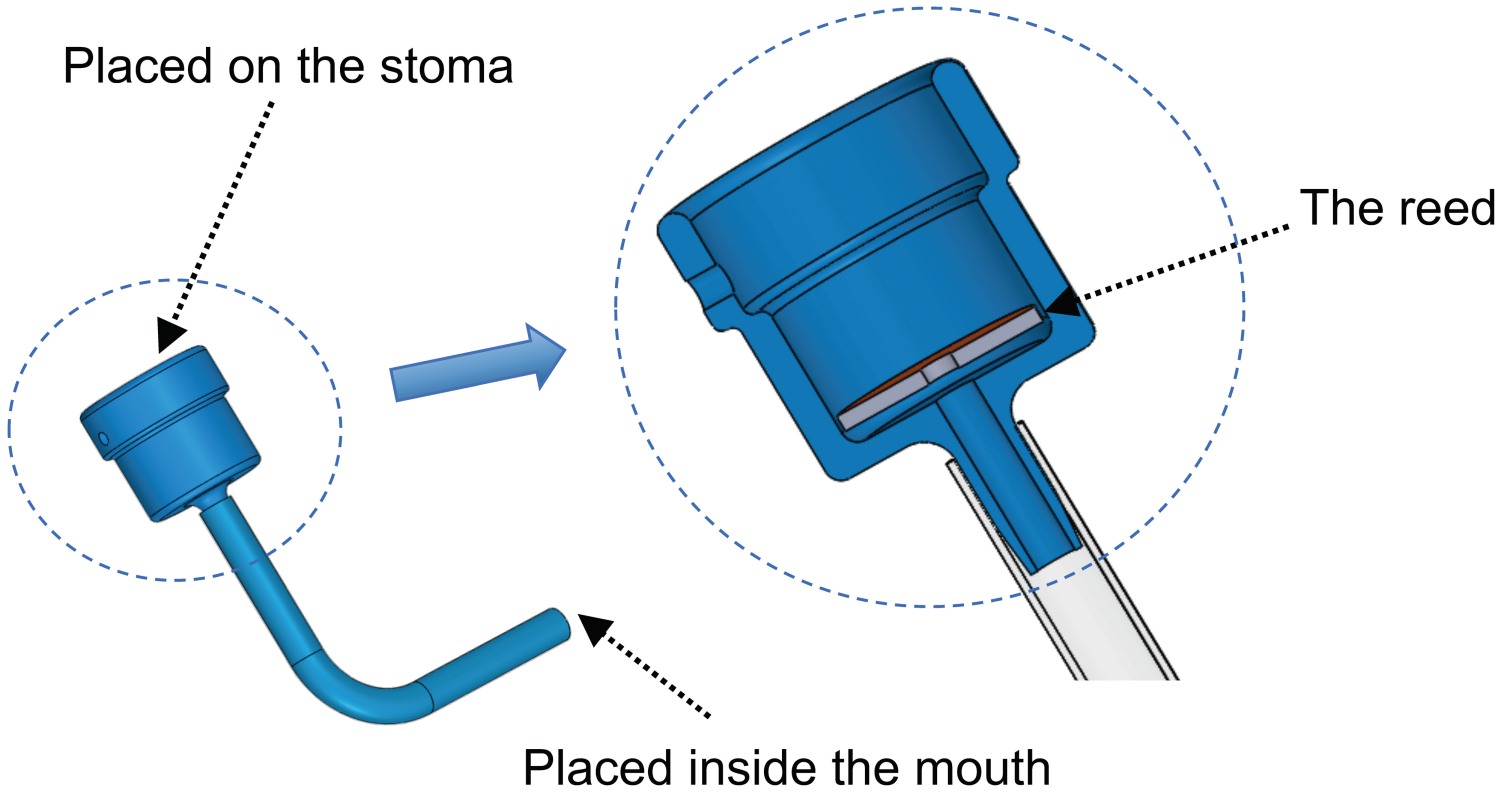
The DSP-8 Pneumatic larynx used in the trials.

The PAL device used in this study is the DSP-8 (Fig 3). It has a rectangular reed element vibrating inside a cylindrical cavity and demonstrates a voice onset/offset hysteresis effect similar to the vocal folds [37] when used in speech. To provide a precise model of the onset/offset control of this PAL which can be also extended to other PAL designs with different shapes of the reed (such as the Tokyo artificial larynx [21]), a numerical optimization approach is proposed. Such optimization is specifically advantageous as the mechanical attributes and the elasticity of the PAL reed seems to change over time and the parameters of a hysteresis model to describe the onset/offset control need to be updated.

A bi-state hysteresis model *H*(*ψ*,∅,*P*_*g*_) is proposed to be adapted to the time-varying attributes of the onset/offset control of this PAL source. In this model, *P*_*g*_(*t*), the pressure inside the source that drives the vibrations at time *t*, is the rectified difference between stoma *P*_*s*_(*t*) and intra-oral *P*_*m*_(*t*) pressure values. The rectified difference (half brackets) is used in (1) since the source will not generate any voice for negative values of the driving pressure.

$$P_{g}\left(t\right)=\left\lfloor P_{s}\left(t\right)-P_{m}\left(t\right)\right\rfloor$$ (1)

The hysteresis function *H*(*ψ*,*P*_*g*_) (2) with *ψ =* {*θ*_*L*_,*θ*_*H*_} has an onset threshold: *θ*_*H*_ and offset threshold: *θ*_*L*_ (*θ*_*H*_ > *θ*_*L*_).

$$H\left(t\right)=H\left(\psi ,P_{g}\left(t\right)\right)=\{\begin{array}{cc} 1 & where\;P_{g}>\theta_{H}\\ 0 & where\;P_{g}<\theta_{L}\\ H\left(\psi ,P_{g}\left(t-1\right)\right) & else \end{array}$$ (2)

The hysteresis function output, *H*(*ψ*,*P*_*g*_(*t*)), is smoothed using three time delays, for attack, release, and hold, ∅ = {*T*_*A*_, *T*_*R*_, *T*_*H*_} (in milliseconds) to from *H*_*s*_(∅,t) in (3). When an onset (offset) instance is detected by (2) an attack (release) counter, *C*_*A*_ (*C*_*R*_) is initiated to keep track of the onset (offset) time. The smoothed Hysteresis output is then defined as *H*_*s*_(t) = *H*_*s*_(∅,t) [45]:

$$H_{s}\left(\emptyset ,t\right)=\{\begin{array}{ccc} T_{A}*H_{s}\left(t-1\right)+\left(1-T_{A}\right)*H\left(t\right) & if\;C_{R}>T_{H}\;and & H_{s}\left(t-1\right)\leq H\left(t\right)\\ \begin{array}{c} \;H\left(t-1\right)\\ T_{R}*H_{s}\left(t-1\right)+\left(1-T_{R}\right)*H\left(t\right) \end{array} & \begin{array}{c} if\;C_{R}\leq T_{H}\\ if\;C_{R}>T_{H}\;and \end{array} & \begin{array}{c} H_{s}\left(t-1\right)>H\left(t\right) \end{array}\\ H\left(t-1\right) & \;if\;C_{A}\leq T_{H} & \end{array}$$ (3)

The smoothed model (3) becomes more robust to noise when *P*_*g*_ approaches the onset and offset thresholds by delaying the decision by increasing the hold time *T*_*H*_. The main task of the modeling is then to optimize the five parameters of the onset and offset thresholds (*θ*_*H*_, *θ*_*L*_) and time delays (*T*_*A*_, *T*_*R*_, *T*_*H*_) when the PAL is used in speech.

## Experimental framework

### Pre-clinical trials of controlling a pneumatic Bionic Voice source onset and offset

The parameters of the model (2, 3) were optimized and its predictions were verified against recordings of the mechanical PAL device through pre-clinical trials. The trials were supported by an ethics approval from the Sydney Local Health District ethics committee, Royal Prince Alfred Hospital zone (protocol number X14-0276, HREC/14/RPAH/362). One laryngectomy patient was enrolled following his expression of interest to the public advertisement of the research. The recruitment continued from January 2015 to September 2018 and written consent was obtained. The participant was a proficient user of the DSP-8 PAL device (Fig 3). The use of only one patient is consistent with the aim of the trials to achieve a precise numerical model of the mechanical PAL device rather than study patients’ interaction with the device.

### The dataset

The laryngectomy patient sat in a quiet room and used the PAL source as his voice prosthesis to speak, while his supra-glottal and subglottal pressures were recorded simultaneously. Recordings of respiration (the patient’s stoma and his intra-oral pressure) and speech were performed at 1 kHz and 48 kHz respectively. The respiration recordings were manually labelled to identify onset/offset instances of the source during speech. For verification, the performance of the model (2, 3) in estimating the onset/offset was evaluated against hand-labelled PAL recordings. The criterion for evaluation has been the correlation coefficient between the estimated and target voiced/unvoiced labels. To elicit words and sentences, standard speech tokens used in the evaluation of intelligibility of dysfunctional voice were used [46–48]. The spoken phrases were the rainbow passage [49] (first paragraph) at slow and normal conversational speech rates, the Diagnostic Rhyme Test (DRT) [50], Modified Rhyme Test (MRT) and the Phonetically Balanced (PB) word list [51]. The length of each recording was 45 seconds. Overall, more than five hours (20,000 seconds) of data were collected. An anonymized dataset underlying the results of this study is available as a Supporting Information File (S1 File).

## Implementation details and results

### Pre-processing

When generating a voice for the patient, the PAL source creates air pressure vibrations which are picked up by the pressure sensors. This contaminates the patient’s recorded stoma and intra-oral pressures. To estimate the voice onset/offset from the respiratory data to control the Pneumatic Bionic Voice source in the absence of the PAL, a pre-processing step is required to remove the PAL source vibrations. Considering $P_{s}{}_{{}_{raw}}\left(t\right)$, $P_{m}{}_{{}_{raw}}\left(t\right)$ as the raw recorded stoma and the intra-oral pressures respectively, a low-pass Butterworth filter (with a cut-off frequency of 70 Hz) is applied to these signals to calculate *P*_*s*_(t) and *P*_*m*_(t) in (1).

### Threshold optimisation on offline data

**Fig 4:**
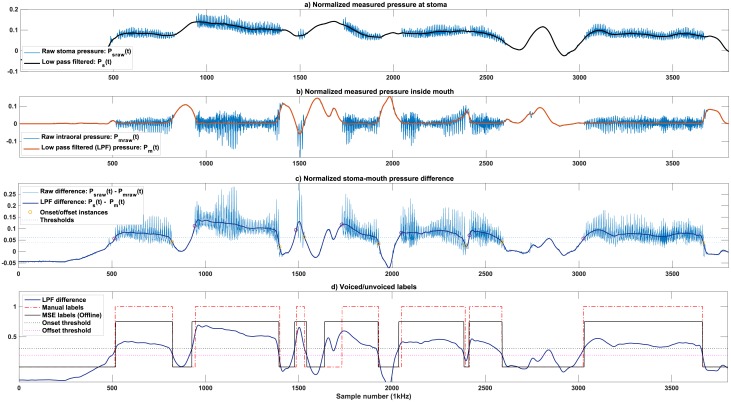
Threshold optimisation on the offline recorded data. The laryngectomy patient used the PAL to generate continuous speech. a, b) Pre-processing of raw stoma and mouth pressures result *P*_*s*_(*t*), *P*_*m*_(*t*), c) The low-pass filtered pressure difference (*P*_*g*_ = *P*_*s*_(*t*)—*P*_*m*_(*t*)) as the input to the hysteresis model (solid line), threshold values (dashed lines) and the onset/offset instances (circles), d) comparison of the MSE labels of the offline PAL model with the manual.

Two scenarios (an offline and a concurrent situation) were considered for finding the parameters of the hysteresis model (2, 3). Fig 4 shows the threshold optimization for the offline scenario. The preprocessing (low-pass filtering) of raw stoma and mouth pressure recordings is depicted in Fig 4a and 4b. The calculated difference between the low-pass filtered pressure of stoma and mouth, *P*_*s*_(t), *P*_*m*_(t) in (1) was the input to the hysteresis model (Fig 4c). The parameters of the model were optimized for each recording of 45 seconds. The attack, release and hold times (*T*_*A*_, *T*_*R*_, *T*_*H*_) were initially set to 1*ms* for all recordings to minimize the response delay of the controller at 1kHz. The two thresholds (*θ*_*H*_, *θ*_*L*_) were then optimized to minimize the Mean Square Error (MSE) between the manual-labels of each recording of 45 seconds with the model predictions. The search range for (*θ*_*H*_, *θ*_*L*_) was modified for each recording: using the manual labels, the set of values of *P*_*g*_ in (1) at which a voice transitions from voiced to unvoiced or vice versa were selected, and their median and Mean Absolute Deviation (MAD) were calculated. For each parameter (*θ*_*H*_, *θ*_*L*_), the range of median ± 3 MAD was then divided into 25 segments, and the MSE search was then performed on the resulting 625 (25×25) points. The calculated thresholds together with the pre-set values of (*T*_*A*_, *T*_*R*_, *T*_*H*_) provided a sufficiently low MSE error so further fine-tuning of (*T*_*A*_, *T*_*R*_, *T*_*H*_) was not needed. Fig 4d shows the offline MSE threshold fitting.

When fitted individually for each recording, the average correlation coefficient of the predicted and manual voiced/unvoiced labels is 98.45 ±0.54% on the recorded dataset. Offline MSE results are promising, but the approach is computationally expensive and needs the full length (45 seconds) of the recording to maintain its accuracy. In addition, the elasticity of the source changes, resulting in variations of *θ*_*H*_ and *θ*_*L*_ between different recordings of the PAL source. However, it is safe to consider *θ*_*H*_, *θ*_*L*_ constant for the full length of each recording (45s). The attack, release and hold times (∅ = {*T*_*A*_, *T*_*R*_, *T*_*H*_}) remained consistent along the entire database.

### Threshold optimisation on concurrent data

To overcome the shortcomings of the offline MSE approach, a concurrent scenario was proposed to minimize the length of data required for estimating the thresholds of each recording. The concurrent scenario was designed for online evaluation of the model against the PAL when the patient is using the PAL to speak. The concurrent approach (which may not be necessarily in real-time) has to adapt to the changes in PAL hysteresis thresholds as the new recording data is accumulated. Thus, it can no longer use a manual labelling of the data as opposed to the offline mode.

**Fig 5:**
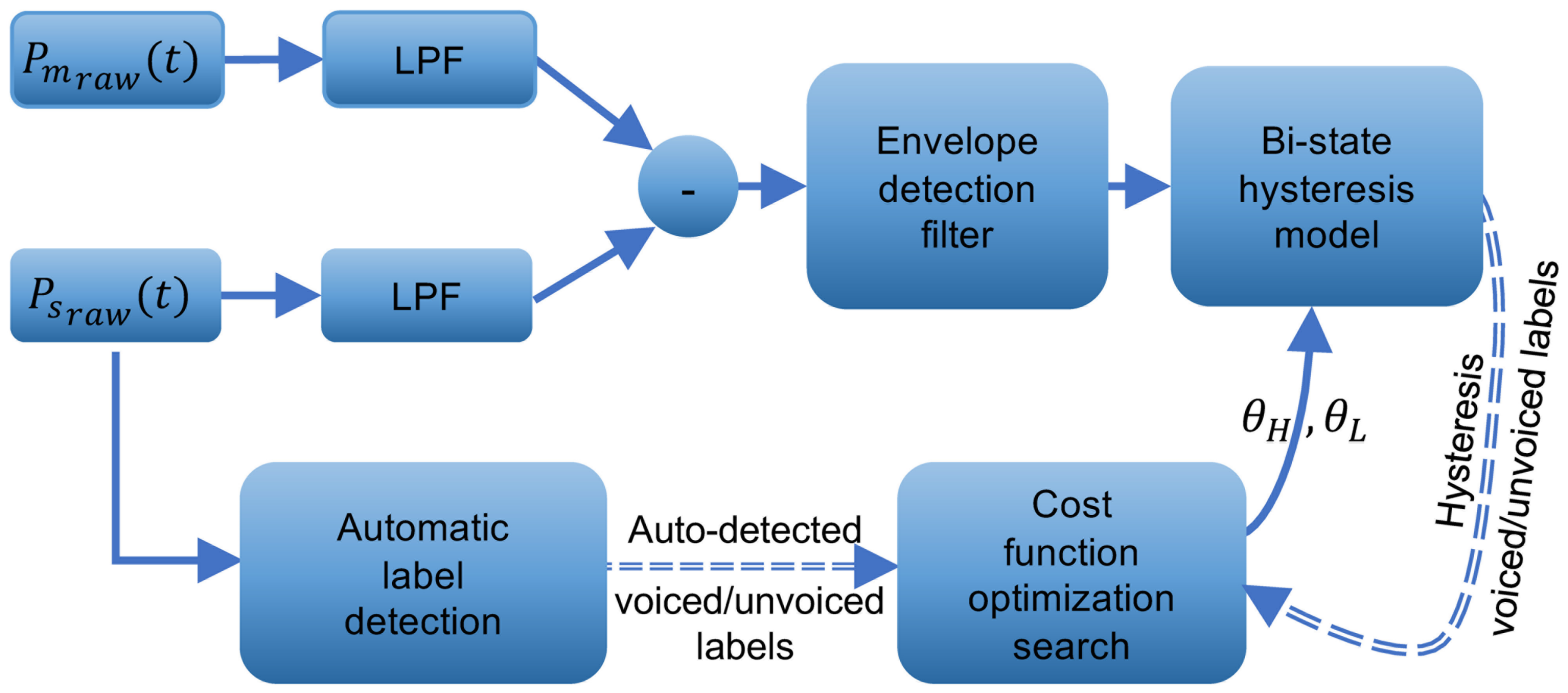
Block diagram of the concurrent threshold optimisation.

The block diagram of the concurrent method is reflected in Fig 5. The system uses a low-pass filtering of the pressure values *P*_*s*_(*t*), *P*_*m*_(*t*) (as described in the Pre-processing section) to provide input to the hysteresis model. In a parallel pathway, an auto label detection module is designed to provide the ground truth of the onset/offset labels at each time step. The auto-labels are detected using the unfiltered PAL recording of the stoma $P_{s}{}_{{}_{raw}}\left(t\right)$ (which is contaminated by the vibrations of the source). With the ground truth established, an optimization algorithm adjusts the model thresholds by minimizing a cost function as a function of the thresholds.

#### Automatic voiced/unvoiced label detection

**Fig 6:**
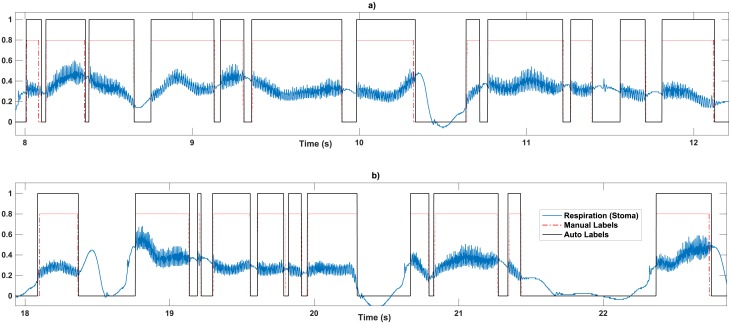
Auto-label detection for two samples of recording of $P_{s}{}_{{}_{raw}}$ in continuous speech. a, b) The patient is reading the rainbow passage. The time axis shows the speed of onset and offset occurrence in speech. The auto-labeller keeps up with the accuracy with conversational speech.

Since the vocal tract acts as a resonant cavity, the raw intra-oral pressure of the mouth $P_{m}{}_{{}_{raw}}\left(t\right)$ will maintain resonances even after the PAL source relinquishes vibrations. Hence detecting the onset/offset labels automatically is more precise from the raw stoma recording $P_{s}{}_{{}_{raw}}\left(t\right)$ (Fig 5). $P_{s}{}_{{}_{raw}}\left(t\right)$ shows pseudo harmonic oscillations added to a slow-varying envelope (Fig 6). The auto-label detection removes this slow-varying envelope from the signal. Applying a moving average filter with a 20-sample window to $P_{s}{}_{{}_{raw}}\left(t\right)$ results in the slow-varying envelope $ma_{Ps}{}_{{}_{raw}}\left(t\right)$. Next, the difference signal *d*_*t*_ is calculated (4) which shows a pseudo harmonic oscillation pattern around zero when the PAL is generating voice.

$$d_{t}=P_{s}{}_{{}_{raw}}\left(t\right)-ma_{Ps}{}_{{}_{raw}}\left(t\right)$$ (4)

To estimate the onset and offset, the moving standard deviation of the rectified difference ${\tilde{d}}_{t}$ is defined where *w*_*t*_ is the moving average window centered at time t.

$$v_{t}=\sqrt{1/w_{t}\sum_{i=t-\frac{w_{t}}{2}}^{t+\frac{w_{t}}{2}}{\left({\tilde{d}}_{i}–{\bar{d}}_{i}\right)}^{2}}$$ (5)

The difference signal *d*_*t*_ oscillates in voiced speech, so the moving standard deviation *v*_*t*_ shows a bi-modal histogram divided by a threshold. In the unvoiced speech, *v*_*t*_ falls in the lower amplitude range of the histogram, moving towards the larger amplitudes for voiced speech. The adaptive histogram thresholding by Otsu et al. [52] is performed to determine the point to divide the *v*_*t*_ histogram into voiced/unvoiced regions. The auto-thresholding method clusters amplitudes of *v*_*t*_ in the histogram into two voiced/unvoiced classes. The class with values higher than the Otsu’s optimal threshold *v*_*t*_ is labelled voiced, and the other unvoiced.

The advantage of the auto-labelling method is that the histogram is dynamic, changing over time as the PAL pressure data is accumulated and the Otsu thresholding [52] is updated over time. This makes the auto-labeler similarly strong in estimating voices/unvoiced instances at continuous speech (Fig 6). When applied to the training dataset, the method provides a high matching of 98.67 ± 0.58% with manual labels for isolated words and 98.2 ±0.9% for the recordings of the rainbow passage at a conversational speech rate.

#### Concurrent optimization of the thresholds

Starting from the beginning of the recording and without prior knowledge about the recorded data, at time steps of *t* = *T*, the optimization of a cost function *f*_*t*_(*θ*_*H*_, *θ*_*L*_) (6) is performed and the thresholds of the hysteresis model (2, 3) are updated. The output of the model has to match PAL ground-truth labels *L*_*t*_ ∈ [0,1], which are calculated through automatic label detection (Fig 5). The cost function of this optimization, *f*_*t*_(*θ*_*H*_, *θ*_*L*_), is defined as the variance of the weighted error between estimated onset/offsets of the hysteresis model *H*(*ψ*, *P*_*g*_(*t*)) and the ground truth (*L*_*t*_).

$$f_{t}\left(\theta_{H},\;\theta_{L}\right)={\text{Var}}_{t=1:T}\left[w_{t}\left(L_{t}-H\left(\psi ,P_{g}\left(t\right)\right)\right)\right]$$ (6)

The error is weighted by *w*_*t*_, i.e., the rectified driving pressure of the PAL, giving more significance to errors observed in larger values of the driving pressure *P*_*g*_(*t*).

$$w_{t}=P_{g}\left(t\right)=\left\lfloor \;P_{s}\left(t\right)-P_{m}\left(t\right)\right\rfloor$$ (7)

The optimization algorithm searches for the threshold values *θ*_*H*_, *θ*_*L*_ to minimize *f*_*t*_(*θ*_*H*_, *θ*_*L*_) at each time step. To facilitate the convergence of the algorithm and reduce the execution time, the history of ground truth labels *L*_*t*_ is used to limit the range of values searched for (*θ*_*H*_, *θ*_*L*_) at each time step. Despite being bistable, if the hysteresis system (2, 3) detects onset at *P*_*g*_(*t*) = *θ*_*H*0_, it will remain in a stable “on” state for increased values of the driving pressure. These values (*P*_*g*_(*t*) > *θ*_*H*0_ and ∇*P*_*g*_(*t*) > 0) should be ruled out from the search domain of the upper threshold *θ*_*H*_ for the next time-step. A median of the elimination candidates is chosen to limit the upper bound of the search domain. For the lower threshold *θ*_*L*_ (which has a smaller dynamic range), the search range is proven sufficient to be limited to {0, median (*θ*_*Lt*_)} where *θ*_*Lt*_ are the calculated lower thresholds of previous time steps.

**Fig 7:**
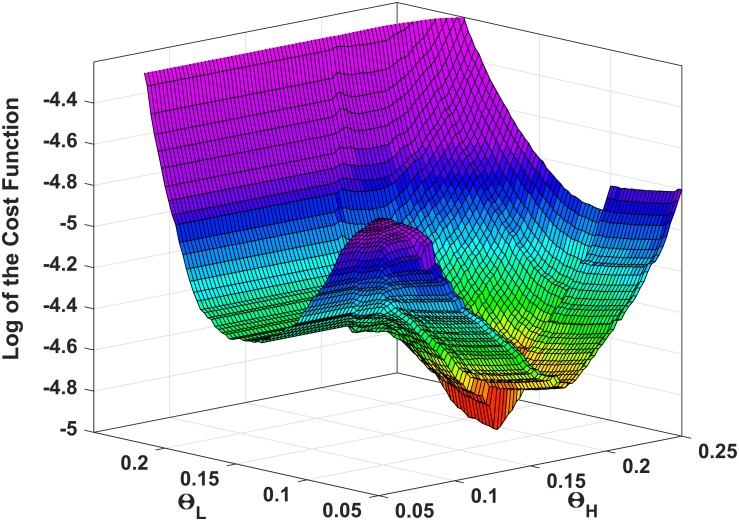
The logarithmic error, cost function *f*_*t*_(*θ*_*H*_, *θ*_*L*_), calculated over the search domain for *θ*_*H*_, *θ*_*L*_ for the dataset.

The cost function *f*_*t*_(*θ*_*H*_, *θ*_*L*_) (6) is non-smooth, discrete and non-differentiable so derivative based approaches may not necessarily converge during optimization. Fig 7 explains the case for convergence of an optimization algorithm in the search domain to minimize *f*_*t*_(*θ*_*H*_, *θ*_*L*_). As observed in this figure, the variations of cost function *f*_*t*_(*θ*_*H*_, *θ*_*L*_) in the recorded dataset with changing thresholds show a global minimum. Yet, the presence of local optima supports the choice of non-gradient based or heuristic optimization techniques. Accordingly, two direct search methods are employed for threshold optimization which do not require any derivative information (explicit or implicit) from *f*_*t*_(*θ*_*H*_, *θ*_*L*_). These are the pattern search [53] and the Nelder–Mead method [54]. The Nelder–Mead method is a widely used multidimensional minimization and is inherently unconstrained. The search domain of the pattern search, however, was bounded as described before.

In both approaches the optimization starts from initial values of the thresholds *θ*_*H*0_ and *θ*_*L*0_ and recursively updates these, using the thresholds values derived at a previous step. At each iteration step (*t*), the pattern search algorithm places a stencil (pattern) centred around the values of the thresholds derived in iteration (*t*−1) in the *R* = {*θ*_*H*_, *θ*_*L*_} domain. This pattern includes a set of search directions to cover the points adjacent to the center. If the cost function value *f*_*t*_(*θ*_*H*_, *θ*_*L*_) is decreased in any of these directions, the pattern is moved to the new center point, otherwise, the stencil size is reduced. The pattern search is terminated when the iteration error is less than the desired accuracy, or the available number of iterations has expired.

**Fig 8:**
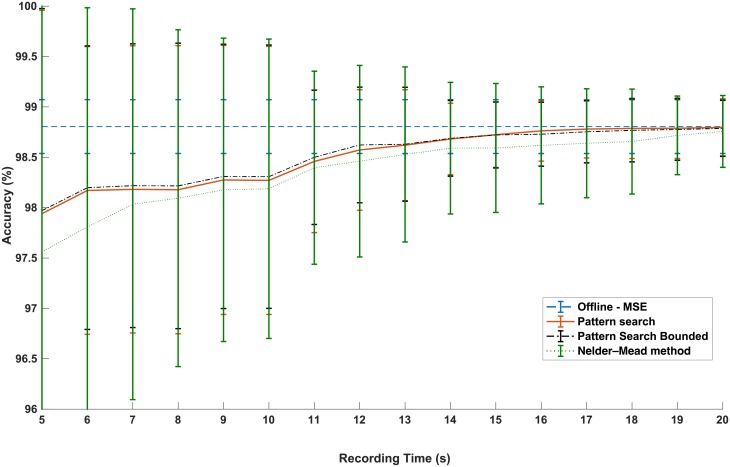
The average accuracy of the hysteresis model in matching manual labels when the hysteresis thresholds are concurrently adapted through three different optimization methods. The bars show the variance of the accuracy for 24 isolated and continuous recordings of 45*s* each. The results are compared against the offline MSE thresholds driven for the full length of 45*s* of each recording.

Fig 8 demonstrates the convergence behavior of the methods when applied to 24 isolated and continuous speech recordings of 45 seconds sampled at 1kHz with a total of 1,080,000 decision points. The accuracy is measured by the percentage of correctly estimated onset samples over the total number of samples. The thresholds have been updated at intervals of T = 1*s*. To avoid any confusion, the reported accuracy is against original manual labels in this figure. As observed in Fig 8, the overall accuracy of the optimization method for pattern search and bounded pattern search is better than the unconstrained Nelder–Mead method with bounded pattern search slightly higher than pattern search. The algorithm needs at least 5 seconds of recording of the patient at 1kHz (using the PAL source to speak) to reach 98 ± 2% of accuracy. However, to reduce the standard deviation of the error for all recordings, 10 seconds of recorded data is needed to limit the variation of the accuracy to remain close to 98.3±1% approaching to 98.7 ± 0.4 at 15 seconds.

**Fig 9:**
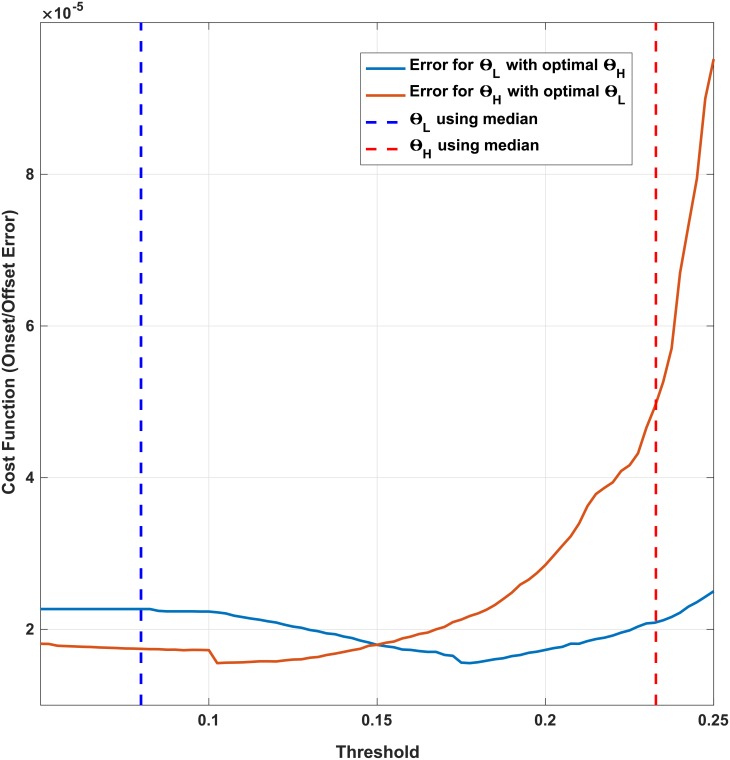
The cost function (error) vs. *θ*_*H*_ and *θ*_*L*_, when the other threshold value is set to optimal. The median values of the pressure amplitude *P*_*g*_(*t*) at onset and offsets are plotted as dashed lines as a rough estimate of *θ*_*H*_, *θ*_*L*_ respectively.

Fig 9 shows a two-dimensional variation of the cost-function *f*_*c*_(*θ*_*H*_, *θ*_*L*_) when the thresholds *θ*_*H*_, *θ*_*L*_ vary around the global optimum. Curves are constructed by first finding the global optimum for both thresholds and then varying each while keeping the other one fixed at its optimal value. A reverse of this strategy has been adopted in the pattern search algorithm to derive the thresholds by optimizing one of the thresholds first and next using its value to derive the optimal value for the other.

### Real-time performance of the voice onset/offset control

To assess the real-time performance, the control algorithm of the Pneumatic Bionic Voice prosthesis (with fixed thresholds) was implemented in parallel with a hardware emulation of the PAL. The PAL hardware emulation was implemented using an Arduino Uno^™^ which streamed out the previously recorded pressure values of the PAL (raw pressures of the mouth and stoma) at 1kHz. The manual labels of the respiration data were loaded to the Arduino^™^ to generate a beep sound in the voice onset states. The Pneumatic Bionic Voice control was implemented using C++ and compiled on a Mac computer with 64 GB RAM and a 3.5 GHz 6 core Intel^™^ Xeon E5 processor and similarly generated a sine wave sound whenever a voice onset was detected in the real-time streamed respiration data. The two audio outputs (from the PAL emulator and the Bionic Voice control algorithm) were isolated and simultaneously recorded in two separate channels of an audio stereo recorder. The measured delay between Bionic Voice and the PAL emulator varied in the range of 5-17*ms* with an average of 10*ms* which was consistent between the onset and offset.

## Discussion

This study defines a new framework for using PAL as an excellent reference for designing a Pneumatic Bionic Voice prosthesis. As the first step, a precise model of the onset and offset control of a Pneumatic Bionic Voice prosthesis is provided with an accuracy of 98.45 ± 0.54% when compared to the PAL performance in a mixed (continuous and isolated) speech database. The onset and offset thresholds, mainly depend on the physical attributes of the PAL reed. However, these may also be speaker-dependent as each speaker uses the PAL in a different range of respiration pressure values. As the attributes of the reed change with time, threshold determination will be session-dependent as well. The fact that the optimal thresholds *θ*_*H*_, *θ*_*L*_ of the offline scenario remain constant in the duration of each session (of 45 seconds) enables source implementation with fixed values of these optimal thresholds. Having uniform thresholds allows the system to perform faster in real-time. Nevertheless, a concurrent threshold optimization is also implemented to enable online evaluations of the source against changes of the PAL attributes over time.

In terms of an intuitive pneumatic control of a voice prosthesis, this study complements Takahashi et al. [55] who implemented a real-time voicing control of an Electrolarynx which terminated the voice after observing a peak in the intra-oral pressure. The concept [55] is similarly valid for the PAL as an increase in the intraoral pressure decreases the driving pressure of a PAL and leads to a voice offset. The real-time voicing control approach of Takahashi et al. [55], has a delay of less than 20*ms*. The system was tested on one laryngectomy patient and reported to correctly detect 90% of unvoiced consonants, improving the misidentification of patient’s voiced/unvoiced stop consonants by 50% [56].

The other significant research direction in intuitive control of an artificial voice source is the myoelectric Bionic Voice prostheses. These are essentially motor control prostheses similar to a Bionic Arm, in which the intended motor function is voice generation. Goldstein et al. [57, 58] established the concept of using surface electromyography (sEMG) of neck strap muscles as a reliable indicator of the voice onset and macro-variations of the pitch of the voice. Fuchs et al. [59] improved these results using a database of sEMG/speech recordings of 19 minutes of phonetically balanced sentences of healthy male and female subjects. With an approach similar to the benchmark study of Goldstein et al. [58], they reached an average onset/offset error range of 9.5 ±5.6% and 6.6 ±4.7% for the female and male subjects respectively. However, the original algorithm [58] provided an accuracy significantly lower than this for laryngectomy patients [46, 58].

Using myoelectric Bionic Voice prostheses to control the voice onset/offset faces two major challenges ahead. The first is accessing neck strap muscles to control the prosthesis since these muscles are normally excised at the time of laryngectomy to minimize the risk of cancer spread [60]. Heaton et al. [61] have demonstrated the feasibility of preserving these muscles although this will require a modification of the standard laryngectomy surgical procedure. Stepp et al. [62] demonstrated the utilization of other residual face/neck muscles for non-invasive myoelectric control of a voice prosthesis, obviating the need for modification of the standard laryngectomy surgical procedure. However, these alternative muscles have not yet provided a reliable substitute for the neck straps in terms of controlling voice onset/offset and the pitch [62].

The second challenge is the significantly larger time scales of controlling muscle contraction/release compared to the real-time control of voice. A myoelectric controller for Bionic Arm can have a reaction delay of up to 300*ms* before being perceived as sluggish by the user [63]. A window size of 150-250*ms* is recommended as optimal when segmenting the sEMG data to control a Bionic Arm in a simple two-class task [64, 65], resulting in an optimal (minimum) controller delay of 100-125*ms* [64, 66]. These are much larger than the voice onset and offset time frames of continuous speech where the delay between the start of a voiceless consonant and the start of voicing for the next vowel is in the range of 10-70*ms* [55, 67–69]. The inability to match this temporal resolution will translate to the voicing of unvoiced phonemes, i.e., the largest source of reduced intelligibility of EL speech [70–72]. Meeting these time limits has been particularly challenging in the myoelectric Bionic Voice when a subject is asked to actively control voice termination by relaxing their neck muscles at the end of a phrase [46, 73] (leading to large voice termination delays of 400- 700*ms* for healthy subjects [74] and 1120-1970*ms* for the laryngectomy participant [46]). Myoelectric Bionic Voice sources, however, seem to be reliable controllers of the pitch (macro-prosody) of the voice which works on a larger time scale at sentence level [74].

These two challenges make the Pneumatic Bionic Voice prosthesis a convenient substitution for the myoelectric Bionic Voice prostheses in terms of onset/offset control. 1) It is a non-invasive, prosthesis which does not require modifications in laryngectomy surgery, 2) In terms of the prosthesis controller delay, the PAL already provides voice onset/offset control in slow and fast speech rates with high intelligibility scores [6–13, 21, 22]. The authors have also achieved a 10*ms* average delay in a real-time implementation of Pneumatic Bionic Voice onset/offset control when compared to the PAL. A direct comparison is not possible due to variations between recorded data sets. However, the precision and speed of the Pneumatic Bionic Voice on the large recorded corpus seem to be uniquely ahead of previous intuitive voice prostheses which enabled a laryngectomy patient to directly control the onset/offset of the voice in continuous speech in real-time [46, 55, 75].

It is worth mentioning that, despite its high quality and the ability to provide pitch variations at syllable level [8, 9], the PAL device (Fig 3) used in this study as the ground truth is a simplified version of an ideal PAL (where a mechanical replica of the vocal folds substitutes the PAL’s simple reed). Hence following an ideal PAL as the reference may improve the results of this study in terms of estimating a speaker’s intended voice onset/offset and the pitch. In addition, even in an ideal configuration, the PAL will lack the potential to modulate the macro-prosody (pitch variation at sentence level). The respiratory control (of the subglottal pressure) may need to be combined with laryngeal control (of the tension of vocal folds) to enable modulating macro-prosody [76]. Meltzner et al. [4] have reported that natural modulation of the pitch is the most influential attribute to improve the quality of speech after laryngectomy [4, 77]. This limitation seems to be the strength of the Myoelectric Bionic Voice solutions [78, 79] and potentially ties the two research directions of Pneumatic and Bionic Voice prostheses together to provide an intuitive control over the onset/offset and the pitch of the voice.

## Conclusion

The PAL can be considered as a simple model of the human larynx with a fixed pair of vocal folds driven exclusively by the variations of the intraoral and subglottal pressure values and without any neural/neuro-muscular input from the missing larynx. The quality of PAL speech is comparable to the existing gold standard of TE voice prostheses and far better than the Electrolarynx [6–12]. The traditional PAL also holds a significant advantage over the existing gold standard as being non-invasive.

These advantages advocate defining a new pathway in designing Pneumatic Bionic Voice prosthesis as electronic adaptations of the PAL. This study aims to be the first in this direction and provides a precise model that describes the PAL voice onset/offset control with a low computational cost suitable for real-time implementations. The next step for the authors is to combine this solution with a PAL pitch modulation model in real-time and evaluate the quality of the resulting speech against the PAL and the existing gold standard.

## Supporting information

S1 FileThis is the dataset of recorded intra-oral and stoma pressure values, manually labelled for voiced/unvoiced detection.The zip archive contains anonymized recordings of time-aligned intra-oral and stoma pressure ($P_{m}{}_{{}_{raw}}\left(t\right)$, $P_{s}{}_{{}_{raw}}\left(t\right)$ respectively), with their voice/unvoiced labels (*L*_*t*_) as the ground truth.(ZIP)
